# Gene modules associated with breast cancer distant metastasis-free survival in the PAM50 molecular subtypes

**DOI:** 10.18632/oncotarget.7774

**Published:** 2016-02-27

**Authors:** Rong Liu, Wei Zhang, Zhao-Qian Liu, Hong-Hao Zhou

**Affiliations:** ^1^ Department of Clinical Pharmacology, Xiangya Hospital, Central South University, Changsha 410008, P. R. China; ^2^ Institute of Clinical Pharmacology, Central South University; Hunan Key Laboratory of Pharmacogenetics, Changsha 410008, P. R. China

**Keywords:** breast cancer, distant metastasis-free survival, gene expression profiling, PAM50 subtype

## Abstract

To identify PAM50 subtype–specific associations between distant metastasis-free survival (DMFS) in breast cancer (BC) patients and gene modules describing potentially targetable oncogenic pathways, a comprehensive analysis evaluating the prognostic efficacy of published gene signatures in 2027 BC patients from 13 studies was conducted. We calculated 21 gene modules and computed hazard ratios (HRs) for DMFS for one-unit increases in module score, with and without adjustment for clinical characteristics. By comparing gene expression to survival outcomes, we derived four subtype-specific prognostic signatures for BC. Univariate and multivariate analyses showed that in the luminal A subgroup, E2F3, PTEN and GGI gene module scores were associated with clinical outcome. In the luminal B tumors, RAS was associated with DMFS and in the basal-like tumors, ER was associated with DMFS. Our defined gene modules predicted high-risk patients in multivariate analyses for the basal-like (HR: 2.19, p=2.5×10^−4^), luminal A (HR: 3.03, p=7.2×10^−5^), luminal B (HR: 3.00, p=2.4×10^−10^) and HER2+ (HR: 5.49, p=9.7×10^−10^) subgroups. We found that different modules are associated with DMFS in different BC subtypes. The results of this study could help to identify new therapeutic strategies for specific molecular subgroups of BC, and could enhance efforts to improve patient-specific therapy options.

## INTRODUCTION

Breast cancer (BC) is a biologically and clinically heterogeneous disease with diverse morphologies, molecular features, and clinical behaviors. Clinicopathologic characteristics such as clinical TNM stage at diagnosis, histologic grade, lymph node involvement and estrogen receptor (ER) and human epidermal growth factor receptor 2 (HER2) statuses have been associated with BC prognosis. Gene expression studies have shown that different molecular BC subtypes exhibit different characteristics and prognoses [1, 2]. Tumor subtypes based on the PAM50 classifier have distinct prognoses, and respond differently to systemic therapy [3–5]. Further, expression signatures for genes such as Gene70 [6], Gene76 [7], Grade Index (GGI) [8] and OncotypeDx [9] have been studied to help identify low-risk patients who are unlikely to benefit from systemic adjuvant therapy, while still correctly identifying high-risk patients [6–11].

Several gene signatures have been identified to describe oncogenes such as RAS, E2F3, SRC, MYC and β-catenin [12], as well as activation of insulin-like growth factor 1 (IGF1) [13], and the AKT/mammalian target of rapamycin (mTOR) [14] and mitogen-activated protein kinase (MAPK) [15] pathways. Other signatures have identified PIK3CA mutations [16], and deficiencies in PTEN [17]. Chemotherapy sensitivity in different BC subtypes is altered based on activation of different oncogenic pathways [18], and Desmedt, *et al.* reported that BC survival depended on ER and HER2 status [4]. However, it is still unknown whether patients within different PAM50 subtypes have different DMFS based on activation of different biological process and oncogenic pathways.

To identify robust, PAM50 subtype–specific associations between DMFS and gene modules describing biologically relevant, potentially targetable oncogenic pathways and prognosis signatures, a comprehensive systematic analysis evaluating the prognostic efficacy of 21 published gene signatures in 2027 BC patients from 13 studies [16, 19–30] was conducted. In this pooled in-silico study, we also investigated whether modules were associated with DMFS beyond clinical characteristics in each molecular subtype. Furthermore, we wanted to confirm previous findings from small sample studies on the association between DMFS and specific pathways, such as AKT-MTOR and RAS [31], and extend our analysis to PAM50 subtypes. This study expands on the analysis conducted by Desmedt, *et al.* [4] in four ways. First, ten more oncogenic pathways were evaluated in our study. Second, only datasets generated using the Affymetrix U133 plus 2.0 or U133A platforms were utilized in our study, while the datasets used in Desmedt, *et al*. [4] were gathered from different platforms, possibly resulting in heterogeneity [32]. The final distinction is that we not only explored the associations between gene modules and BC patient survival within each PAM50 subtype, but we also identified four subtype-specific prognosis modules that could predict high-risk of distant metastasis.

## RESULTS

### Identification of PAM50 subgroups

The clinicopathological characteristics of the 2027 BC patients included in the study are listed in Table 1. Out of the 2027 samples, we classified 554 as luminal A, 774 as luminal B, 129 as HER2+, 440 as basal-like and 130 as normal-like. There are differences between subtypes based on PAM50 with regard to DMFS. The survival probability for DMFS (Supplementary Figure S1) of the basal-like subtype was lower than the luminal A and luminal B groups (Hazard ratio (HR)=0.37, P < 2.0×10^−16^ for luminal A; HR=0.80, P = 1.5×10^−2^ for luminal B). However, no survival difference was found between the basal-like and HER2+ subgroups (HR=1.07, P= 0.65).

**Table 1:** Patient characteristics from the gene expression data sets

Characteristic	GSE7390	GSE9195	GSE16446	GSE45255	GSE20685	GSE6532		GSE11121	GSE12093	GSE2603	GSE25066	GSE42568	GSE19707	GSE12276
Sample size	75	59	107	135	238	190	37	140	111	80	508	104	39	204
Age,years														
<=50	51	2	107	54	154	42	0	0	0	0	264	27	18	0
>50	24	57	0	81	84	148	37	0	0	0	244	77	21	0
Unknown	0	0	0	0	0	0	0	140	111	80	0	0	0	204
Histologic grade														
1	12	8	2	16	0	30	3	16	0	0	32	11	0	0
2	27	17	19	49	0	114	18	98	0	0	180	40	9	0
3	36	19	81	67	0	22	9	26	0	0	259	53	29	204
Unknown	0	15	5	3	238	24	7	0	111	80	37	0	1	
cN														
positive	0	27	59	44	0	44	24	0	0	0	351	59	25	0
negative	75	32	48	91	0	142	13	140	0	0	157	45	11	0
Unknown	0	0	0	0	238	0	0	0	111	80	0	0	3	204
ER														
Positive	44	59	0	88	144	160	37	90	67	46	300	68	14	129
Negative	31	0	107	47	94	30	0	50	44	34	208	36	25	75
HER2														
Positive	9	4	42	61	64	16	7	2	1	19	6	41	31	129
Negative	66	55	65	74	174	174	30	138	110	61	502	63	8	75
DMFS event	50	10	24	32	82	66	21	41	18	27	111	48	17	185
DMFS (year):mean±sd	4.9±2.8	7.1±2.0	3.0±1.5	4.2±2.0	5.8±2.9	5.4±2.9	5.4±2.9	5.6±2.7	6.5±2.1	5.0±2.2	3.0±1.6	4.5±2.7	3.2±2.4	2.2±1.8
Platform	GPL96	GPL570	GPL96	GPL96	GPL96	GPL96	GPL570	GPL96	GPL96	GPL96	GPL96	GPL570	GPL570	GPL570
Reference	Desmedt et al. [19]	Loi et al. [16]	Desmedt et al. [20]	Nagalla et al. [21]	Kao et al. [22]	Loi et al. [23]		Schmidt et al. [24]	Zhang et al. [25]	Minn et al.[26]	Hatzis et al. [27]	Clark et al. [28]	Sircoulomb et al. [29]	Bos et al. [30]

Abbreviations: cN, clinical nodal status; ER, estrogen receptor; FISH, fluorescent in situ hybridization; HER2, human epidermal growth factor receptor 2.

ER status determined by IHC for patients or inferred by single ESR1 gene mRNA expression.

HER2 status determined by IHC/FISH for patients or inferred by single HER2 gene mRNA expression.

### Pair-wise gene module correlations

**Figure 1:**
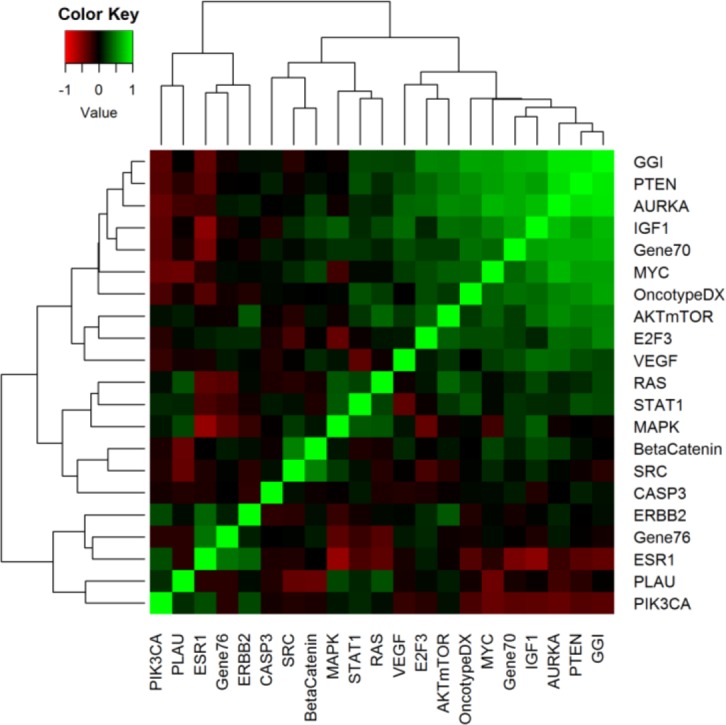
A heat map presents pair-wise correlations between different modules in the cohort with 2027 BC patients The cells are colored on the basis of Pearson's correlation coefficient values, with green and red indicative of positive and negative correlations, respectively.

Pair-wise correlations between 21 gene modules for the pooled population are depicted in Figure 1 (Pearson's coefficient correlations are listed in Supplementary Table S3). GGI, a gene signature that mainly measures tumor proliferation, was highly correlated with Oncotype DX, Gene70, modules describing proliferation (AURKA) and PTEN loss, and MYC and IGF1 pathway activation.

### Gene modules associated with DMFS

**Figure 2:**
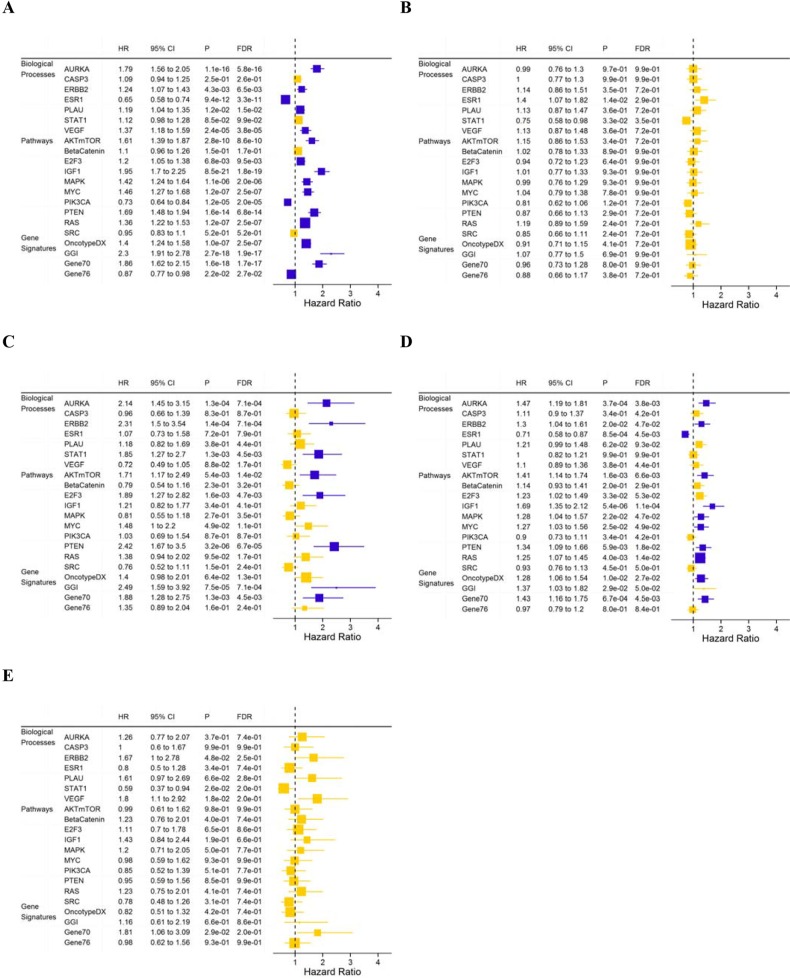
Hazard ratios for DMFS for one-unit increase in module score in a Cox regression model with the data set as stratum indicator for all patients **A.** and the basal-like **B.** luminal A **C.** luminal B **D.** and HER2+ **E.** subtypes. Horizontal bars represent the 95% CIs, the dimension of the square in inverse proportion to the SE of HRs; Modules with significant association (FDR<0.05) are shown in orange. FDR, false discovery rate.

Univariate associations between gene modules and DMFS were analyzed for all patients and PAM50 subtypes (Figure 2). High module scores of AURKA, Gene70, AKTmTOR, HER2 receptor signaling and PTEN loss were associated with poor DMFS in the luminal A and luminal B subtypes, but not in the basal-like and HER2+ subtypes. Additionally, a significant reduction in DMFS was associated with low ER signaling, PIK3CA and Gene76 module scores, and high PLAU, VEGF, E2F3, IGF1, MAPK, MYC, RAS, OncotypeDX and GGI module scores for the entire cohort (Figure 2A). Luminal A tumors with high STAT1, E2F3 and Gene70 module scores had poor DMFS (Figure 2C); luminal B tumors with high activation of IGF1, MAPK, MYC, and OncotypeDX gene modules and low activation of the ESR1 module had poor DMFS (Figure 2D). However, there is no module significantly associated with DMFS within the basal-like and HER2+ subtypes after FDR adjustment (Figure 2B & 2E).

In a multivariate model including age, histologic grade, node status and treatment, we found that node-positivity; grades 2 and 3, no adjuvant treatment and chemotherapy were all associated with poor DMFS for the entire cohort (Table 2). Additionally, older patients (>=50 years) with basal-like tumors who received chemotherapy, had grade 2 or 3 luminal A tumors, or had luminal B tumors with positive nodal status had poor DMFS (Table 2). There was no covariate associated with DMFS in the HER2+ subtype.

**Figure 3:**
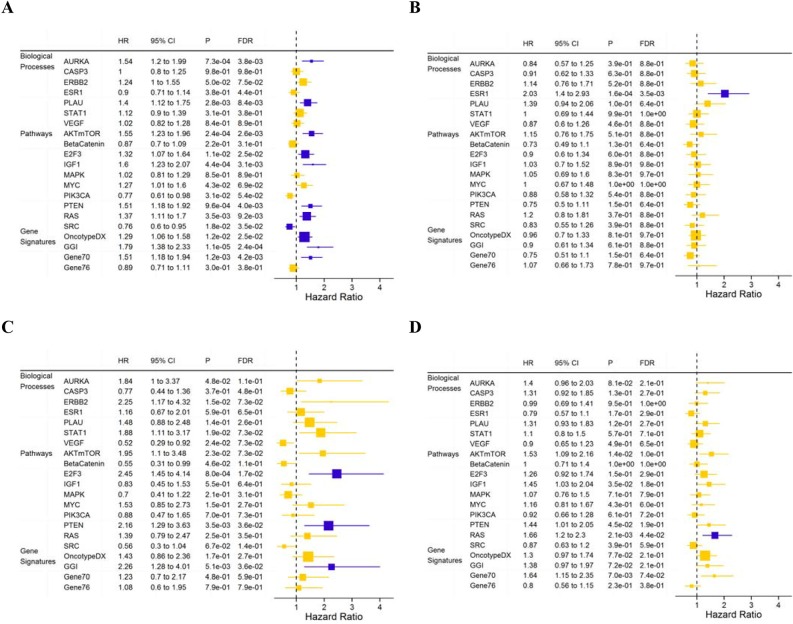
Hazard ratios for DMFS for one-unit increase of module score in a Cox regression model with the data set as stratum indicator for all patients after adjustment for clinical nodal status, histologic grade and treatment **A.** basal-like subtype after adjustment for age and treatment **B.** luminal A subtype after adjustment for histologic grade **C.** and luminal B subtype after adjustment for clinical nodal status **D.** Horizontal bars represent the 95% CIs, the dimension of the square in inverse proportion to the SE of HRs; Modules with significant association (FDR<0.05) are shown in orange. FDR, false discovery rate.

We also analyzed the prognostic values of the gene expression modules adjusted for the clinical parameters selected above (Figure 3). For the entire cohort, low SRC signaling and high AURKA, AKTmTOR, E2F3, IGF1, PTEN-loss, RAS, OncotypeDX, GGI and Gene70 module scores were associated with poor DMFS (Figure 3A). In the luminal A subpopulation (n = 554), the E2F3 (adjusted HR=2.45; 95% CI: 1.45-4.14; P = 8.0×10^−4^, FDR=1.7×10^−2^), PTEN loss (HR=2.16; 95% CI: 1.29-3.63; P = 3.5×10^−3^, FDR=3.6×10^−2^) and GGI (HR=2.26; 95% CI: 1.28-4.01; P = 5.1×10^−3^, FDR=3.6×10^−2^) modules were associated with DMFS (Figure 3C). In the luminal B tumors (n = 774), only the RAS module was associated with DMFS (HR=1.66; 95% CI: 1.20-2.30; P = 2.1×10^−3^, FDR=4.4×10^−2^) in the multivariate model (Figure 3D); in the basal-like tumors (n=440), only the ER module was associated with DMFS (HR=2.03; 95% CI: 1.40-2.92; P = 1.6×10^−4^, FDR=3.5×10^−3^) (Figure 3B).

**Table 2:** Multivariate Cox regression model for DMFS in patients with complete clinical and genomic data according to breast cancer subtypes

Cohort	Characteristic	HR	95% CI	P-value
All patients(n=1045)	cN (Positive vs. Negative)	1.74	1.32–2.31	1.03×10^−4^
	Histologic grade (2 vs. 1)	2.81	1.57–5.03	4.82×10^−4^
	Histologic grade (3 vs.1)	3.82	2.11–6.89	9.17×10^−6^
	Treatment (chemo vs. both)	1.97	1.34–2.89	5.72×10^−4^
	Treatment (endo vs. both)	1.79	0.75–4.23	1.88×10^−1^
	Treatment (no adjuvant vs. both)	5.20	1.15–23.49	3.22×10^−2^
Basal (n=276)	Age (>=50 vs. <50)	1.55	1.00–2.42	4.96×10^−2^
	Treatment (chemo vs. both)	3.14	1.12–8.76	2.91×10^−2^
LumA(n=261)	Histologic grade (2 vs. 1)	2.24	0.96–5.25	6.31×10^−2^
	Histologic grade (3 vs. 1)	5.89	2.04–16.99	1.02×10^−3^
LumB(n=362)	cN (Positive vs. Negative)	1.69	1.07–2.67	2.41×10^−2^

Abbreviations: In the Cox regression model, the clinicopathologic factors, age, histologic grade, node status and treatment were included as covariates, and only the significant (P<0.05) factors are shown in this table.

cN, clinical nodal status; chemo, chemotherapy; endo, endocrine therapy; both, both endocrine therapy and chemotherapy; no adjuvant, did not receive systematic adjuvant.

### Development of a prognostic survival module within each subtype

For each BC PAM50 molecular subtype, we identifed subtype-specific prognostic signatures. To identify the prognostic gene candidates, we calculated HRs in relation to DMFS for each gene. Then, 20 candidate modules with the most significant 10 to 200 prognostic gene candidates were determined (e.g., module 1 with the most significant 10 prognostic genes, module 2 with the most significant 20 prognostic genes… module 20 with the most significant 200 prognostic genes.), and corresponding module scores were calculated. Finally, HRs of DMFS for one unit increase in module score were computed at the univariate and multivariate levels, and the module with highest HRs was identified. From this process, modules with 70, 60, 20 and 150 genes for basal-like, luminal A, luminal B and HER2+ subtypes were identified (Supplementary Figure S2). The HRs of DMFS for the four subtype-specific modules were listed in Supplementary Table S6. Compositions and weights of the modules are listed in Supplementary Table S2.

**Figure 4:**
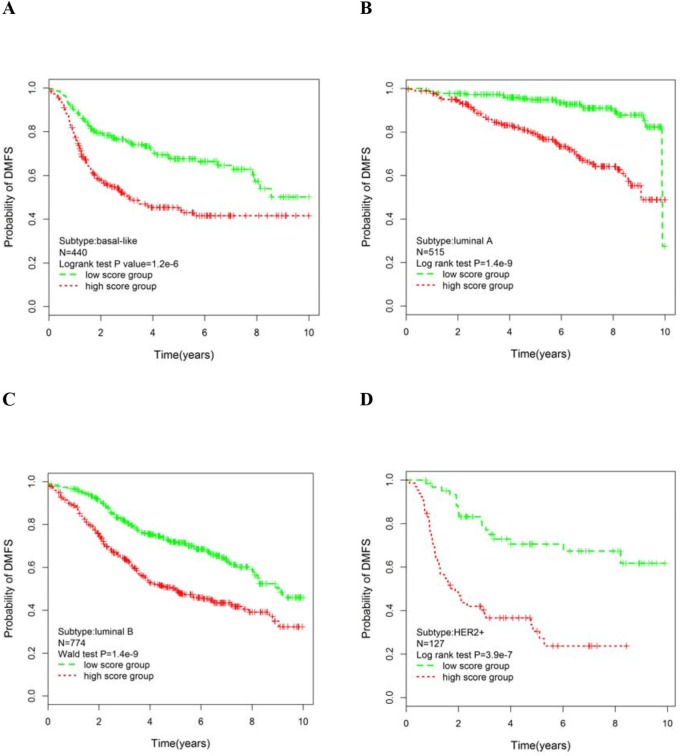
Kaplan-Meier curves of significant module scores in the univariate analysis for the PAM50 molecular subgroups Patients were grouped according to the median of the module score: basal subgroup **A.** luminal A subgroup **B.** luminal B subgroup **C.** and HER2+ subgroup **D.** P-values were obtained from the log-rank test.

The patients were divided into high and low risk using the median cutoff of the subtype-specific module scores, and survival risk prediction analysis was performed. To evaluate patient prognosis, Kaplan-Meier survival curves were drawn and the log-rank test showed significant differences in DMFS for the basal-like (p=1.2×10^−6^; Figure 4A), luminal A (p=1.4×10^−9^; Figure 4B), luminal B (p=1.4×10^−9^; Figure 4C) and HER2+ (p=3.9×10^−7^; Figure 4D) subtypes.

GO biological process analysis showed that the basal-like, luminal A and HER2+ specific modules contained genes enriched in the immune response, cell cycle process and response to chemical stimulus, respectively (Table 3). Genes in the luminal B module were not significantly associated with any specific processes.

**Table 3:** List of the GO term in the significant DAVID functional cluster for basal-like, luminal A and Her2+ subtype-specific modules

Module	Biological process term	Gene count	%	P-value
Basal-like specific module	immune response	18	24	1.45×10^−8^
	immune system process	20	27	1.08×10^−7^
	antigen processing and presentation	5	7	6.43×10^−4^
	response to stimulus	29	39	1.65×10^−3^
	taxis	5	7	7.00×10^−3^
Luminal A specific module	cell cycle	15	25	5.13×10^−7^
	cell cycle phase	11	18	2.22×10^−6^
	M phase	10	17	2.77×10^−6^
	microtubule-based process	9	15	3.63×10^−6^
	cell cycle process	12	20	5.20×10^−6^
Luminal B specific module	response to chemical stimulus	26	17	1.43×10^−4^
	regulation of myeloid cell differentiation	6	4	3.98×10^−4^
	regulation of biological quality	27	18	4.84×10^−4^
	regulation of multicellular organismal process	20	13	6.32×10^−4^
	regulation of cell proliferation	17	11	1.70×10^−3^

## DISCUSSION

To identify biological processes, oncogenic pathways and prognosis signatures associated with DMFS in PAM50 BC subtypes, an extensive analysis of gene expression data generated from 2027 BC patient samples was conducted. We have shown that molecular subtype-specific modules contain distinct prognostic information. Further, we identified four subtype-specific prognosis signatures that successfully predict high-risk BC patients with poor survival outcomes.

In the entire study population, low ER signaling, PIK3CA and Gene76 module scores, and high AURKA, E2F3, IGF1, MYC, PTEN, RAS, OncotypeDX, GGI and Gene70 module scores were associated with poor DMFS. It was previously reported that low ER signaling and high RAS module scores were associated with poor DMFS [31]. Moreover, it is not surprising that the proliferation-related modules (AURKA, OncotypeDX, GGI and Gene70) are involved in BC prognosis, since increased expression of proliferation-related genes was associated with poor outcome [33].

In the luminal A subgroup, the E2F3 and PTEN pathways appeared to predict DMFS both at the univariate and multivariate levels. High E2F3 and PTEN loss module scores were associated with poor outcome. Similarly, Hollern, *et al*. reported that the E2F transcription factors play important roles in regulating tumor development and metastasis in a mouse module of metastatic BC [34]. Schade, *et al*. found that tumors from PTEN-deficient/NIC mice showed histopathological and molecular features of the luminal subtype of primary human BC, and PTEN deficiency in this type of mouse model leads to dramatic acceleration of mammary tumorigenesis and metastasis. Functional studies still need to be conducted to determine whether E2F3 and PTEN loss actually mediate tumorigenesis and metastasis, and affect prognosis of luminal A BC.

Our study also found that in the luminal B subgroup, high expression of the RAS pathway was significantly associated with poor clinical outcome. Zhang, *et al.* reported that RAS GTPase-activating protein SH3 domain-binding protein 1 (G3BP1), an essential RAS mediator, participates in the progression of BC via activation of the epithelial-to-mesenchymal transition, and that it could be a potential therapeutic target for metastatic human BC [35]. In addition, a recent study suggested that RAS signaling activation was a key determinant for metastatic dissemination and was strongly linked to poor survival of luminal BC patients [36].

These findings emphasize the need for additional prognostic markers for molecular subgroups, specifically for the HER2+ and basal-like subgroups, which are associated with limited therapeutic options and poor prognosis. In our integrated study, four subtype-specific prognosis signatures were identified, with some overlaps between the genes within different modules (Supplementary Figure S3). The luminal A-specific gene module contains genes involved in the cell cycle process, which is correlated with clinical outcomes of BC [33]. The HER2+ specific gene module contains genes involved in response to chemical stimulus. It has been reported that an expression signature enriched in response to chemical stimulus was related to acquired anthracycline resistance in human BC cells [37]. The basal-like specific module contained genes enriched in the immune response. Interestingly, Desmedt, *et al*. [4] and Teschendorff, *et al.* [38] reported that immune response might be linked with development of distance metastases of BC. Moreover, tumor-infiltrating lymphocytes, routinely used as immune response markers, are most frequently found in triple-negative BC, and their higher presence at diagnosis is associated with better clinical outcomes after adjuvant chemotherapy in this subtype [39, 40].

There are several caveats to our study. First, as a retrospective study, heterogeneous patient cohorts were included. Second, we did not attempt to identify an optimal cutoff, but rather used a continuous value for the various modules based on their associations with DMFS. Because different gene expression-based platforms and protocols were used in each study, standardization of a module cutoff value may be unreliable. Third, the prognostic discriminative power of our defined four subtype-specific signatures should be validated in prospective clinical trials.

Despite these limitations, our observations may have potential implications for the clinical management of BC. First, we provide additional evidence that different biological processes and oncogenic pathways are associated with DMFS in different BC subtypes. Second, our results generate hypotheses that should be tested in BC subtype–focused trials of targeted agents. Specifically, it may be worth combining RAS pathway-targeted therapeutics, like Mek inhibitors, together with routine therapy for luminal B BC patients. For luminal A subtypes, PTEN loss modules and E2F3 activation are associated with poor DMFS, suggesting that these patients are likely to benefit from poly (ADP-ribose) polymerase (PARP) inhibitors [41]. For the HER2+ subtype, our prognosis signature included genes involved in response to chemical stimulus. As for basal-like subtype tumors, our results emphasize the importance of immune response in relation to DMFS, and suggest that routine assessment and quantification of tumor-infiltrating lymphocytes could provide meaningful prognostic information in a clinical setting.

In conclusion, we show that different BC tumor characteristics significantly influence DMFS of patients in different PAM50 subtypes. Moreover, the four new prognosis signatures developed in this study for different molecular subgroups could help to further advance personalized medicine for BC patients.

## MATERIALS AND METHODS

### Patients and gene expression data

**Figure 5:**
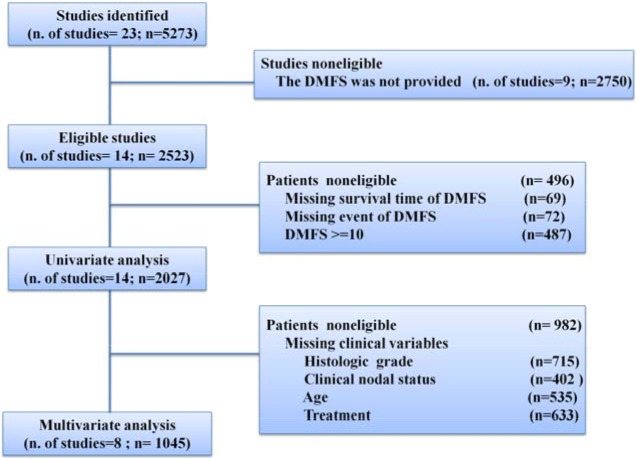
Study flow chart DMFS, distant metastasis free survival.

We searched the Gene Expression Omnibus (GEO) databases (http://www.ncbi.nlm.nih.gov/geo/) using the following key words: breast cancer, GPL96 or GPL570. From all retrieved abstracts, studies that analyzed genome-wide gene expression data generated with Affymetrix U133A or U133Aplus2 gene chips using pretreatment biopsies from BC patients who did not receive primary systemic therapy were identified. Only studies that provided DMFS data were used, and all DMFS data were censored at 10 years. Based on the above criteria, 2027 BC patients from 13 studies [16, 19–30] were selected (Figure 5). Detailed information about each study is provided in Supplementary Table S4.

Normalized gene expression data were downloaded from the GEO data repository with accession numbers: GSE7390 [19], GSE9195 [16], GSE16446 [20], GSE45255 [21], GSE20685 [22], GSE6532 [23], GSE11121 [24], GSE12093 [25], GSE2603 [26], GSE25066 [27], GSE42568 [28], GSE17907 [29] and GSE12276 [30]. The accession numbers were used to name these datasets. Characteristics of the cohorts are summarized in Table 1. We used the probe set 205225_at for ER, 216836_s_at and 234354_x_at for HER2 with Affymetrix U133A and U133Aplus2 platforms, respectively, as previously reported [42]. The cutoffs for ER and HER2 expression were derived from fitting two normal distributions to the observed distribution of expression values for each study separately using the MCLUST function in R. Probes were mapped to gene symbols, and the ones without known gene symbols were filtered. When multiple probes were mapped to the same gene, the average expression of these probes in a particular data set was selected to represent the gene with the collapsedRows function [43] in R.

### Breast cancer molecular subtypes

We assessed the molecular subtypes for each tumor based on the PAM50 algorithm [44] with the “genefu” R package (http://www.bioconductor.org/packages/release/bioc/html/genefu.html). Samples belonging to the basal-like, HER2+, luminal A, and luminal B subtypes were included for subtype-based analysis.

### Module scores

To compute module scores derived from gene signatures for each sample within different datasets, we calculated module score as follows:

$$\text{Module score }=\frac{\sum_{i\in n}w_{i}x_{i}}{\sum_{i\in n}\left|w_{i}\right|}$$

where n is the gene number in a specific module, x_i_ was the expression level of the gene, and gene-specific weights w_i_ were equal to −1 or +1 depending on the direction of their association with the phenotype in the original publication. Each module score was robustly scaled within a study so that the 2.5%, 50% and 97.5% quintiles equaled −1, 0 and +1, respectively, allowing for comparison between different datasets generated with different microarray technologies and normalization procedures. Compositions and weights of the gene modules are provided in Supplementary Table S1.

### Development of molecular subtype-specific prognostic survival modules

For each BC molecular subtype, the following two steps were included in the identification of the subtype-specific prognostic signature: (1) determination of the association between each gene and DMFS and (2) determination of the module with the optimal gene number, with high prognostic prediction ability between gene expression and DMFS. The Cox regression analysis was used to evaluate the association between DMFS and the expression level of each gene after divided by its median; we estimated the HRs of each gene within each dataset separately and combined them using inverse-variance weighting within a fixed effect model. As for prognostic prediction, we ranked the DMFS-associated genes according to their p values of HRs, then defined 20 candidate modules with the number of top ranked prognostic genes:

$$\alpha_{n}=10+(n-1)*10$$

where n was 1 to 20, and computed module scores. As described in the Module scores section, if the combined HR of a specific gene was larger than 1 then its weight was 1, or else its weight was −1. Finally, the module that was most closely correlated with DMFS in univariate and multivariate analyses, that is, with the largest HR value for one unit increase of the module score, was identified.

### Statistical analysis

Statistical analyses were performed using the R statistical software version 3.2.0 [45] with our customized functions. Modeling strategy is described in Supplementary Table S5. Within the entire cohort, we performed pair-wise correlations between the different modules using the Pearson's correlation. All reported p-values were two sided.

### Survival analysis

We considered DMFS as the survival end point. Survival analysis was conducted via the ‘survival’ R package. Survival curves were based on Kaplan-Meier estimates.

To study the univariate association relationships between gene modules and DMFS across different datasets, HRs and 95% CIs for one unit increase in module score were computed using a Cox regression model with the dataset as stratum indicator, allowing for different baseline HRs between different cohorts. This kind of analysis was also performed within PAM50 subtypes.

For multivariate analysis between gene modules and DMFS, a Cox regression model with the independent variables, age (>=50 or <50), clinical nodal status (negative or positive), histologic grade (1, 2 or 3) and treatment (chemotherapy, endocrine therapy, both endocrine therapy and chemotherapy, or no adjuvant therapy) was used to select the covariates significantly related to DMFS (p<0.05) for adjustment. We calculated adjusted HRs for DMFS for one-unit increase in module score with the data sets as stratum indicator.

For multiple module testing adjustment, the false discovery rate (FDR) values were computed with the p.adjust R function with the fdr option.

### Gene ontology and functional analysis

Gene ontology (GO) analyses to test modules for enrichment of genes associated with particular biological processes were done using DAVID (http://david.abcc.ncifcrf.gov/) [46], a web-delivered application that enables the discovery, visualization, and exploration of molecular interaction networks in gene expression data.

## SUPPLEMENTARY FIGURES AND TABLES









## References

[1] Perou CM, Sorlie T, Eisen MB, van de Rijn M, Jeffrey SS, Rees CA, Pollack JR, Ross DT, Johnsen H, Akslen LA, Fluge O, Pergamenschikov A, Williams C, Zhu SX, Lonning PE, Borresen-Dale AL (2000). Molecular portraits of human breast tumours. Nature.

[2] Sotiriou C, Pusztai L (2009). Gene-expression signatures in breast cancer. The New England journal of medicine.

[3] Liedtke C, Mazouni C, Hess KR, Andre F, Tordai A, Mejia JA, Symmans WF, Gonzalez-Angulo AM, Hennessy B, Green M, Cristofanilli M, Hortobagyi GN, Pusztai L (2008). Response to neoadjuvant therapy and long-term survival in patients with triple-negative breast cancer. Journal of clinical oncology.

[4] Desmedt C, Haibe-Kains B, Wirapati P, Buyse M, Larsimont D, Bontempi G, Delorenzi M, Piccart M, Sotiriou C (2008). Biological processes associated with breast cancer clinical outcome depend on the molecular subtypes. Clinical cancer research.

[5] Chia SK, Bramwell VH, Tu D, Shepherd LE, Jiang S, Vickery T, Mardis E, Leung S, Ung K, Pritchard KI, Parker JS, Bernard PS, Perou CM, Ellis MJ, Nielsen TO (2012). A 50-gene intrinsic subtype classifier for prognosis and prediction of benefit from adjuvant tamoxifen. Clinical cancer research.

[6] van't Veer LJ, Dai H, van de Vijver MJ, He YD, Hart AA, Mao M, Peterse HL, van der Kooy K, Marton MJ, Witteveen AT, Schreiber GJ, Kerkhoven RM, Roberts C, Linsley PS, Bernards R, Friend SH (2002). Gene expression profiling predicts clinical outcome of breast cancer. Nature.

[7] Wang Y, Klijn JG, Zhang Y, Sieuwerts AM, Look MP, Yang F, Talantov D, Timmermans M, Meijer-van Gelder ME, Yu J, Jatkoe T, Berns EM, Atkins D, Foekens JA (2005). Gene-expression profiles to predict distant metastasis of lymph-node-negative primary breast cancer. Lancet.

[8] Sotiriou C, Wirapati P, Loi S, Harris A, Fox S, Smeds J, Nordgren H, Farmer P, Praz V, Haibe-Kains B, Desmedt C, Larsimont D, Cardoso F, Peterse H, Nuyten D, Buyse M (2006). Gene expression profiling in breast cancer: understanding the molecular basis of histologic grade to improve prognosis. J Natl Cancer Inst.

[9] Paik S, Shak S, Tang G, Kim C, Baker J, Cronin M, Baehner FL, Walker MG, Watson D, Park T, Hiller W, Fisher ER, Wickerham DL, Bryant J, Wolmark N (2004). A multigene assay to predict recurrence of tamoxifen-treated, node-negative breast cancer. The New England journal of medicine.

[10] van de Vijver MJ, He YD, van't Veer LJ, Dai H, Hart AA, Voskuil DW, Schreiber GJ, Peterse JL, Roberts C, Marton MJ, Parrish M, Atsma D, Witteveen A, Glas A, Delahaye L, van der Velde T (2002). A gene-expression signature as a predictor of survival in breast cancer. The New England journal of medicine.

[11] Volinia S, Croce CM (2013). Prognostic microRNA/mRNA signature from the integrated analysis of patients with invasive breast cancer. Proceedings of the National Academy of Sciences of the United States of America.

[12] Bild AH, Yao G, Chang JT, Wang Q, Potti A, Chasse D, Joshi MB, Harpole D, Lancaster JM, Berchuck A, Olson JA, Marks JR, Dressman HK, West M, Nevins JR (2006). Oncogenic pathway signatures in human cancers as a guide to targeted therapies. Nature.

[13] Creighton CJ, Casa A, Lazard Z, Huang S, Tsimelzon A, Hilsenbeck SG, Osborne CK, Lee AV (2008). Insulin-like growth factor-I activates gene transcription programs strongly associated with poor breast cancer prognosis. Journal of clinical oncology.

[14] Majumder PK, Febbo PG, Bikoff R, Berger R, Xue Q, McMahon LM, Manola J, Brugarolas J, McDonnell TJ, Golub TR, Loda M, Lane HA, Sellers WR (2004). mTOR inhibition reverses Akt-dependent prostate intraepithelial neoplasia through regulation of apoptotic and HIF-1-dependent pathways. Nature medicine.

[15] Creighton CJ, Hilger AM, Murthy S, Rae JM, Chinnaiyan AM, El-Ashry D (2006). Activation of mitogen-activated protein kinase in estrogen receptor alpha-positive breast cancer cells in vitro induces an in vivo molecular phenotype of estrogen receptor alpha-negative human breast tumors. Cancer research.

[16] Loi S, Haibe-Kains B, Majjaj S, Lallemand F, Durbecq V, Larsimont D, Gonzalez-Angulo AM, Pusztai L, Symmans WF, Bardelli A, Ellis P, Tutt AN, Gillett CE, Hennessy BT, Mills GB, Phillips WA (2010). PIK3CA mutations associated with gene signature of low mTORC1 signaling and better outcomes in estrogen receptor-positive breast cancer. Proceedings of the National Academy of Sciences of the United States of America.

[17] Saal LH, Johansson P, Holm K, Gruvberger-Saal SK, She QB, Maurer M, Koujak S, Ferrando AA, Malmstrom P, Memeo L, Isola J, Bendahl PO, Rosen N, Hibshoosh H, Ringner M, Borg A (2007). Poor prognosis in carcinoma is associated with a gene expression signature of aberrant PTEN tumor suppressor pathway activity. Proceedings of the National Academy of Sciences of the United States of America.

[18] Ignatiadis M, Singhal SK, Desmedt C, Haibe-Kains B, Criscitiello C, Andre F, Loi S, Piccart M, Michiels S, Sotiriou C (2012). Gene modules and response to neoadjuvant chemotherapy in breast cancer subtypes: a pooled analysis. Journal of clinical oncology.

[19] Desmedt C, Piette F, Loi S, Wang Y, Lallemand F, Haibe-Kains B, Viale G, Delorenzi M, Zhang Y, d'Assignies MS, Bergh J, Lidereau R, Ellis P, Harris AL, Klijn JG, Foekens JA (2007). Strong time dependence of the 76-gene prognostic signature for node-negative breast cancer patients in the TRANSBIG multicenter independent validation series. Clinical cancer research.

[20] Desmedt C, Di Leo A, de Azambuja E, Larsimont D, Haibe-Kains B, Selleslags J, Delaloge S, Duhem C, Kains JP, Carly B, Maerevoet M, Vindevoghel A, Rouas G, Lallemand F, Durbecq V, Cardoso F (2011). Multifactorial approach to predicting resistance to anthracyclines. Journal of clinical oncology.

[21] Nagalla S, Chou JW, Willingham MC, Ruiz J, Vaughn JP, Dubey P, Lash TL, Hamilton-Dutoit SJ, Bergh J, Sotiriou C, Black MA, Miller LD (2013). Interactions between immunity, proliferation and molecular subtype in breast cancer prognosis. Genome biology.

[22] Kao KJ, Chang KM, Hsu HC, Huang AT (2011). Correlation of microarray-based breast cancer molecular subtypes and clinical outcomes: implications for treatment optimization. BMC cancer.

[23] Loi S, Haibe-Kains B, Desmedt C, Lallemand F, Tutt AM, Gillet C, Ellis P, Harris A, Bergh J, Foekens JA, Klijn JG, Larsimont D, Buyse M, Bontempi G, Delorenzi M, Piccart MJ (2007). Definition of clinically distinct molecular subtypes in estrogen receptor-positive breast carcinomas through genomic grade. Journal of clinical oncology.

[24] Schmidt M, Bohm D, von Torne C, Steiner E, Puhl A, Pilch H, Lehr HA, Hengstler JG, Kolbl H, Gehrmann M (2008). The humoral immune system has a key prognostic impact in node-negative breast cancer. Cancer research.

[25] Zhang Y, Sieuwerts AM, McGreevy M, Casey G, Cufer T, Paradiso A, Harbeck N, Span PN, Hicks DG, Crowe J, Tubbs RR, Budd GT, Lyons J, Sweep FC, Schmitt M, Schittulli F (2009). The 76-gene signature defines high-risk patients that benefit from adjuvant tamoxifen therapy. Breast cancer research and treatment.

[26] Minn AJ, Gupta GP, Siegel PM, Bos PD, Shu W, Giri DD, Viale A, Olshen AB, Gerald WL, Massague J (2005). Genes that mediate breast cancer metastasis to lung. Nature.

[27] Hatzis C, Pusztai L, Valero V, Booser DJ, Esserman L, Lluch A, Vidaurre T, Holmes F, Souchon E, Wang H, Martin M, Cotrina J, Gomez H, Hubbard R, Chacon JI, Ferrer-Lozano J (2011). A genomic predictor of response and survival following taxane-anthracycline chemotherapy for invasive breast cancer. Jama.

[28] Clarke C, Madden SF, Doolan P, Aherne ST, Joyce H, O'Driscoll L, Gallagher WM, Hennessy BT, Moriarty M, Crown J, Kennedy S, Clynes M (2013). Correlating transcriptional networks to breast cancer survival: a large-scale coexpression analysis. Carcinogenesis.

[29] Sircoulomb F, Bekhouche I, Finetti P, Adelaide J, Ben Hamida A, Bonansea J, Raynaud S, Innocenti C, Charafe-Jauffret E, Tarpin C, Ben Ayed F, Viens P, Jacquemier J, Bertucci F, Birnbaum D, Chaffanet M (2010). Genome profiling of ERBB2-amplified breast cancers. BMC cancer.

[30] Bos PD, Zhang XH, Nadal C, Shu W, Gomis RR, Nguyen DX, Minn AJ, van de Vijver MJ, Gerald WL, Foekens JA, Massague J (2009). Genes that mediate breast cancer metastasis to the brain. Nature.

[31] Tobin NP, Harrell JC, Lovrot J, Egyhazi Brage S, Frostvik Stolt M, Carlsson L, Einbeigi Z, Linderholm B, Loman N, Malmberg M, Walz T, Ferno M, Perou CM, Bergh J, Hatschek T, Lindstrom LS (2015). Molecular subtype and tumor characteristics of breast cancer metastases as assessed by gene expression significantly influence patient post-relapse survival. Annals of oncology.

[32] Tan PK, Downey TJ, Spitznagel EL, Xu P, Fu D, Dimitrov DS, Lempicki RA, Raaka BM, Cam MC (2003). Evaluation of gene expression measurements from commercial microarray platforms. Nucleic acids research.

[33] Colozza M, Azambuja E, Cardoso F, Sotiriou C, Larsimont D, Piccart MJ (2005). Proliferative markers as prognostic and predictive tools in early breast cancer: where are we now?. Annals of oncology.

[34] Hollern DP, Honeysett J, Cardiff RD, Andrechek ER (2014). The E2F transcription factors regulate tumor development and metastasis in a mouse model of metastatic breast cancer. Molecular and cellular biology.

[35] Zhang H, Ma Y, Zhang S, Liu H, He H, Li N, Gong Y, Zhao S, Jiang JD, Shao RG (2015). Involvement of Ras GTPase-activating protein SH3 domain-binding protein 1 in the epithelial-to-mesenchymal transition-induced metastasis of breast cancer cells via the Smad signaling pathway. Oncotarget.

[36] Wright KL, Adams JR, Liu JC, Loch AJ, Wong RG, Jo CE, Beck LA, Santhanam DR, Weiss L, Mei X, Lane TF, Koralov SB, Done SJ, Woodgett JR, Zacksenhaus E, Hu P (2015). Ras Signaling Is a Key Determinant for Metastatic Dissemination and Poor Survival of Luminal Breast Cancer Patients. Cancer research.

[37] Lee YS, Ryu SW, Bae SJ, Park TH, Kwon K, Noh YH, Kim SY (2015). Cross-platform meta-analysis of multiple gene expression profiles identifies novel expression signatures in acquired anthracycline-resistant breast cancer. Oncology reports.

[38] Teschendorff AE, Miremadi A, Pinder SE, Ellis IO, Caldas C (2007). An immune response gene expression module identifies a good prognosis subtype in estrogen receptor negative breast cancer. Genome biology.

[39] Loi S, Sirtaine N, Piette F, Salgado R, Viale G, Van Eenoo F, Rouas G, Francis P, Crown JP, Hitre E, de Azambuja E, Quinaux E, Di Leo A, Michiels S, Piccart MJ, Sotiriou C (2013). Prognostic and predictive value of tumor-infiltrating lymphocytes in a phase III randomized adjuvant breast cancer trial in node-positive breast cancer comparing the addition of docetaxel to doxorubicin with doxorubicin-based chemotherapy: BIG 02-98. Journal of clinical oncology.

[40] Adams S, Gray RJ, Demaria S, Goldstein L, Perez EA, Shulman LN, Martino S, Wang M, Jones VE, Saphner TJ, Wolff AC, Wood WC, Davidson NE, Sledge GW, Sparano JA, Badve SS (2014). Prognostic value of tumor-infiltrating lymphocytes in triple-negative breast cancers from two phase III randomized adjuvant breast cancer trials: ECOG 2197 and ECOG 1199. Journal of clinical oncology.

[41] Mendes-Pereira AM, Martin SA, Brough R, McCarthy A, Taylor JR, Kim JS, Waldman T, Lord CJ, Ashworth A (2009). Synthetic lethal targeting of PTEN mutant cells with PARP inhibitors. EMBO molecular medicine.

[42] Gong Y, Yan K, Lin F, Anderson K, Sotiriou C, Andre F, Holmes FA, Valero V, Booser D, Pippen JE, Vukelja S, Gomez H, Mejia J, Barajas LJ, Hess KR, Sneige N (2007). Determination of oestrogen-receptor status and ERBB2 status of breast carcinoma: a gene-expression profiling study. The Lancet Oncology.

[43] Miller J, Cai C, Langfelder P, Geschwind D, Kurian S, Salomon D, Horvath S (2011). Strategies for aggregating gene expression data: The collapseRows R function. BMC Bioinformatics.

[44] Parker JS, Mullins M, Cheang MC, Leung S, Voduc D, Vickery T, Davies S, Fauron C, He X, Hu Z, Quackenbush JF, Stijleman IJ, Palazzo J, Marron JS, Nobel AB, Mardis E (2009). Supervised risk predictor of breast cancer based on intrinsic subtypes. Journal of clinical oncology.

[45] R CT (2014). R: A language and environment for statistical computing. http://wwwr-projectorg/.

[46] Huang da W, Sherman BT, Lempicki RA (2009). Systematic and integrative analysis of large gene lists using DAVID bioinformatics resources. Nature protocols.

