# Supplementary material for: Gene modules associated with breast cancer distant metastasis-free survival in the PAM50 molecular subtypes
Source: Oncotarget. 2016 Feb 27;7(16):21686–98. doi: 10.18632/oncotarget.7774 (PMC5008315; doi:10.18632/oncotarget.7774)
Supplement: Supplementary file 1 [file oncotarget-07-21686-s001.pdf]

Gene modules associated with breast cancer distant metastasis-free survival in the PAM50 molecular subtypes

SUPPLEMENTARY FIGURES AND TABLES

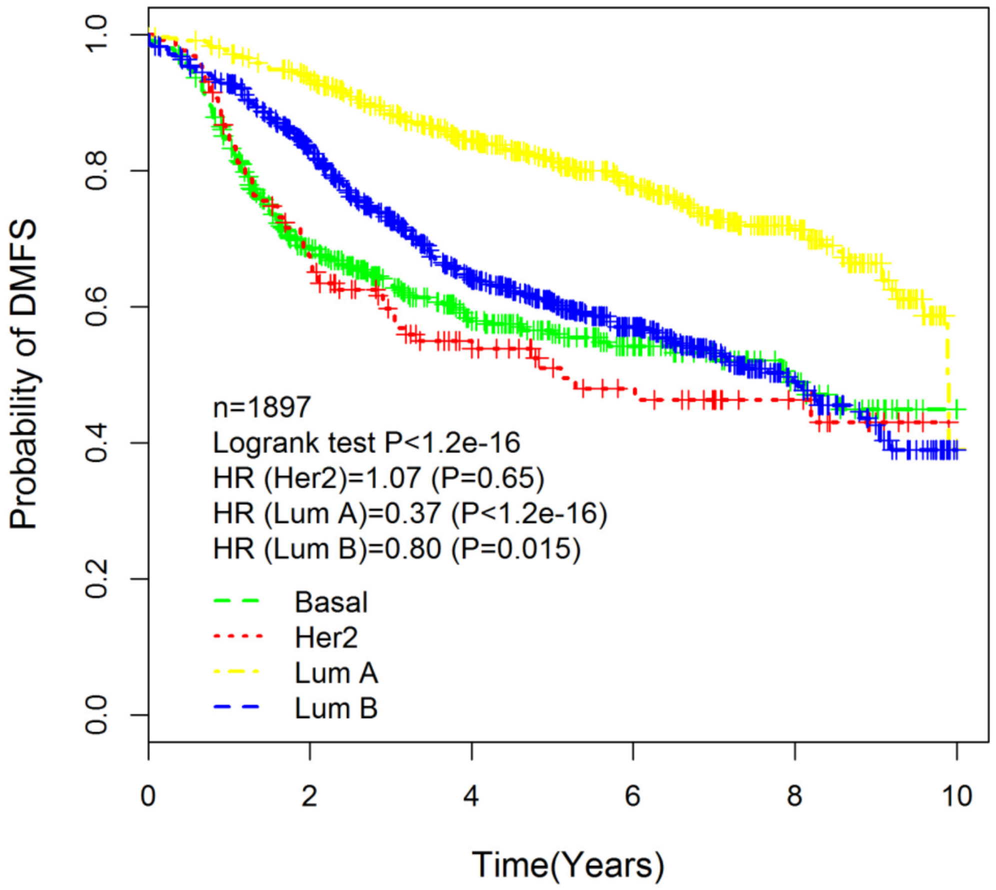

Supplementary Figure S1: Survival curves for untreated patients stratified by PAM50 molecular subtypes.

A

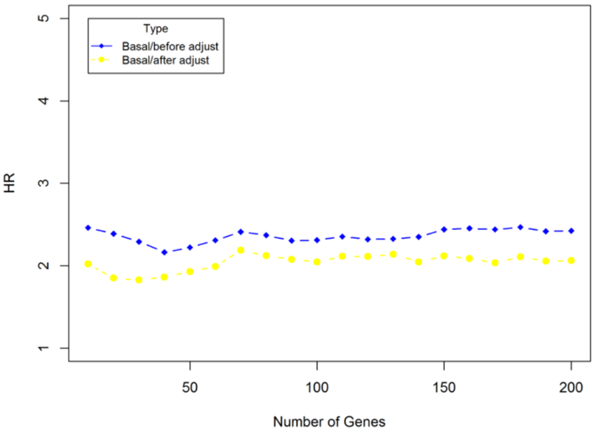

B

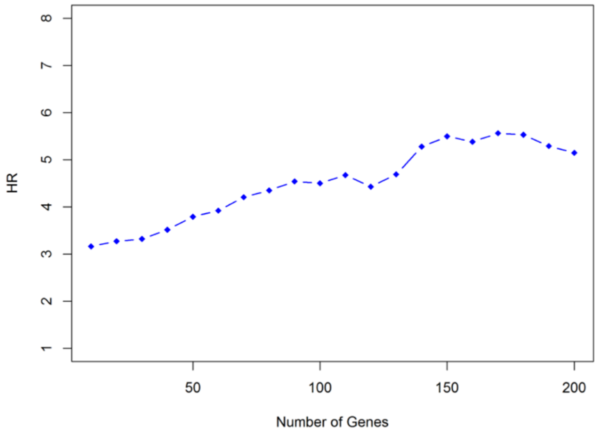

C

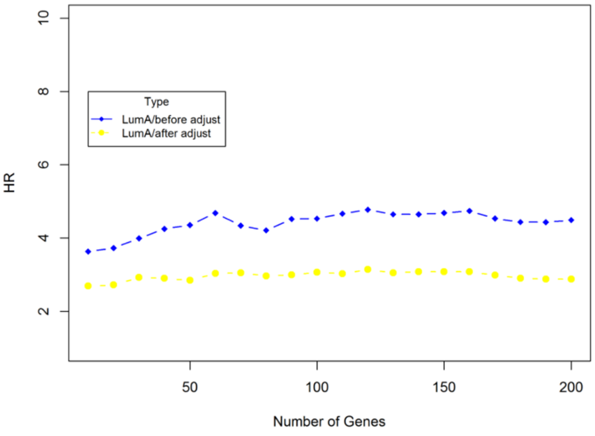

D

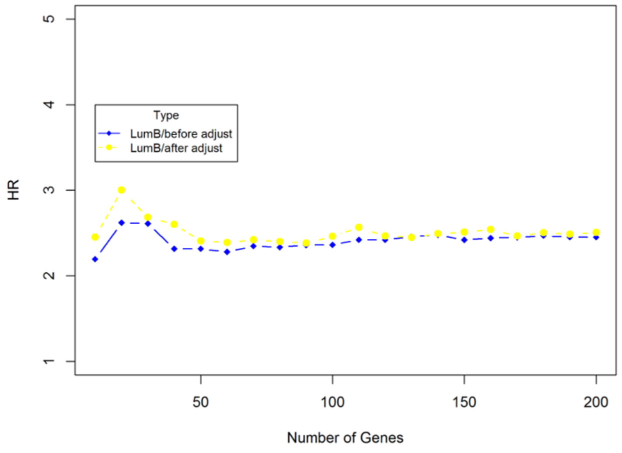

Supplementary Figure S2: Hazard ratios for distant metastasis free survival for high module scores grouped by median in a Cox regression model with the data set as stratum indicator for basal-like A. HER2 positive **B.** luminal A **C.** and luminal B **D.** subtypes for modules with different numbers of significant genes. The covariates used for adjustment were clinical nodal status for the basal subtype, histologic grade for the luminal A subtype, and clinical nodal status for the luminal B subtype.

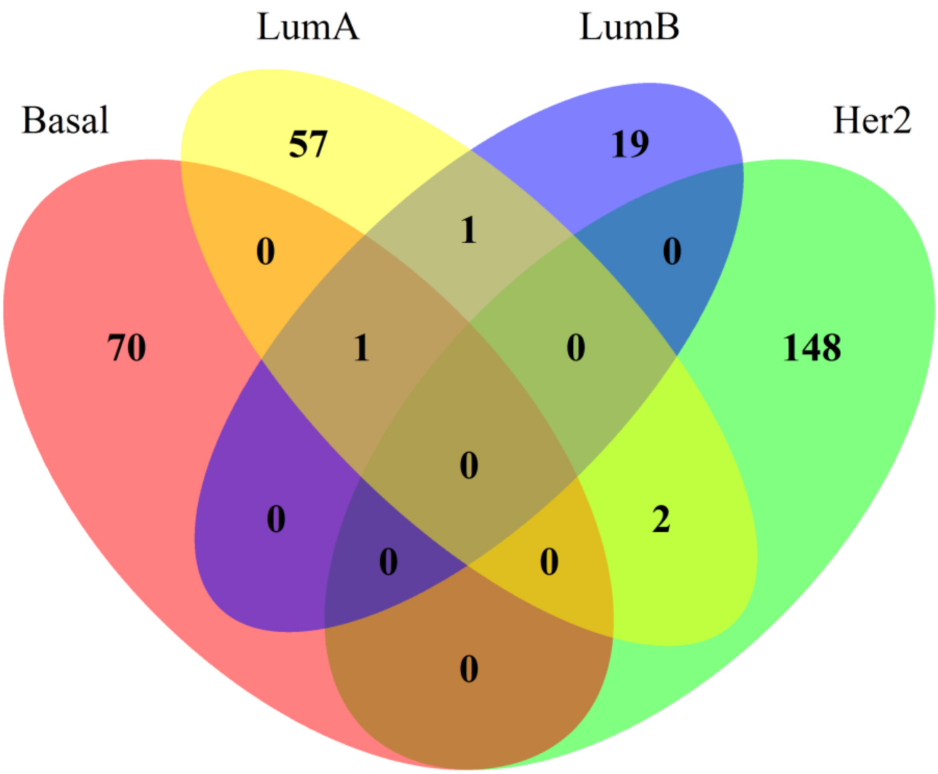

Supplementary Figure S3: Venn diagram showing the overlap of gene lists in our defined subtype-specific modules.

Supplementary Table S1: Summary of gene expression signatures

See Supplementary File 1

Supplementary Table S2: Summary of the subtype-specific gene module compositions and corresponding coefficients

See Supplementary File 2

Supplementary Table S3: Module score pearson correlation coefficients

See Supplementary File 3

Supplementary Table S4: Summary of publically available breast cancer microarray datasets

GEO Number	Platform	Publicyear	Sample size	Treatment	Author	Paper Title	References
GSE9195	GPL570	2008	77	Endocrine therapy	Loi, et al.	Predicting prognosis using molecular profiling in estrogen receptor-positive breast cancer treated with tamoxifen	BMC Genomics 2008
GSE45255	GPL96	2013	139	Endocrine therapy or chemotherapy	Nagalla, *et al.*	Expression Profiles of Breast Tumors from Singapore and Europe	Genome Biol 2013
GSE7390	GPL96	2007	198	No systemic treatment	Desmedt, *et al.*	Strong Time Dependence of the 76-Gene Prognostic Signature	Clin Cancer Res 2007
GSE12093	GPL96	2008	136	Endocrine therapy	Zhang, et al.	The 76-gene Signature Defines High-Risk Patients that Benefit from Adjuvant Tamoxifen Therapy	Breast Cancer Res Treat 2009
GSE25066	GPL96	2011	508	Chemotherapy, ER+ or PR+ patients also received endocrine therapy	Hatzis, et al.	Genomic predictor of response and survival following neoadjuvant taxane-anthracycline chemotherapy in breast cancer	JAMA 2011
GSE11121	GPL96	2008	200	No systemic treatment	Schmidt, *et al.*	The humoral immune system has a key prognostic impact in node-negative breast cancer	Cancer Res 2008
GSE42568	GPL570	2013	121	Unknown	Clarke C, *et al.*	Correlating transcriptional networks to breast cancer survival: a large-scale coexpression analysis	Carcinogenesis 2013

(*Continued*)

GEO Number	Platform	Publicyear	Sample size	Treatment	Author	Paper Title	References
GSE20685	GPL570	2011	327	Unknown	Kao KJ, *et al.*	Correlation of microarray-based breast cancer molecular subtypes and clinical outcomes: implications for treatment optimization	BMC Cancer 2011
GSE12276	GPL570	2009	204	Unknown	Bos PD, et al.	Genes that mediate breast cancer metastasis to the brain	Nature 2009
GSE17907	GPL570	2009	109	Chemotherapy	Sircoulomb F, *et al.*	Genome profiling of ERBB2-amplified breast cancers.	BMC Cancer 2010
GSE2603	GPL96	2005	121	Unknown	Minn AJ et al.	Genes that mediate breast cancer metastasis to lung	Nature 2005
GSE16446	GPL570	2011	120	Chemotherapy	Desmedt C, *et al.*	Multifactorial approach to predicting resistance to anthracyclines	J Clin Oncol 2011
GSE6532	GPL570	2007	87	Endocrine therapy	Loi S, *et al.*	Definition of clinically distinct molecular subtypes in estrogen receptor-positive breast carcinomas through genomic grade	J Clin Oncol 2007
	GPL96	2006	189	Endocrine therapy	Loi S, *et al.*	Definition of clinically distinct molecular subtypes in estrogen receptor-positive breast carcinomas through genomic grade	J Clin Oncol 2007

Supplementary Table S5: Modeling strategy

	Objective	Patients include	Variables included	Result
Univariate	Association between gene module and distant metastasis free survival	All	Module+ strata(dataset)	Figure 3
	Association between gene module and distant metastasis free survival	Pam50 subtypes	Module+ strata(dataset)	Figure 3
Multivariate	Association between gene module and distant metastasis free survival	All	nodal status + grade +module + treatment+ strata(dataset)	Figure 4
	Adjusted association between gene module and distant metastasis free survival	Basal	nodal status +module +strata(dataset)	Figure 4
		Luminal A	grade +module +strata(dataset)	
		Luminal B	nodal status +module + strata(dataset)	

Supplementary Table S6: HRs of DMFS for the four subtype specific modules

Module	Number of genes	Univariate HR		Multivariate HR	
		Value	p-value	Value	p-value
Basal-like specific module	70	2.41	5.4×10^{-10}	2.19	2.5×10^{-4}
Luminal A specific module	60	4.68	1.3×10^{-13}	3.03	7.2×10^{-5}
Luminal B specific module	20	2.62	$<1.2\times10^{-16}$	3.00	2.4×10^{-10}
Her2+ specific module	150	5.49	9.7×10^{-10}	_	_

In the multivariate Cox regression model, hazard ratios for distant metastasis free survival for one-unit increase in module score with the data set as stratum indicator for basal-like subtype after adjustment for age and treatment, luminal A subtype after adjustment for histologic grade, and luminal B subtype after adjustment for clinical nodal status are shown. No significant covariates were found in the multivariate model for Her2+ subtype.
